# Opsonising Antibodies to *P. falciparum* Merozoites Associated with Immunity to Clinical Malaria

**DOI:** 10.1371/journal.pone.0074627

**Published:** 2013-09-09

**Authors:** Danika L. Hill, Emily M. Eriksson, Connie S. N. Li Wai Suen, Chris Y. Chiu, Victoria Ryg-Cornejo, Leanne J. Robinson, Peter M. Siba, Ivo Mueller, Diana S. Hansen, Louis Schofield

**Affiliations:** 1 Walter and Eliza Hall Institute of Medical Research, Parkville, Victoria, Australia; 2 Department of Medical Biology, University of Melbourne, Parkville, Victoria, Australia; 3 Vector Borne Disease Unit, Papua New Guinea Institute of Medical Research, Goroka, Eastern Highlands Province, Papua, New Guinea; 4 Barcelona Center for International Health, University of Barcelona, Barcelona, Spain; Université Pierre et Marie Curie, France

## Abstract

Naturally acquired humoral immunity to the malarial parasite *Plasmodium falciparum* can protect against disease, although the precise mechanisms remain unclear. Although antibody levels can be measured by ELISA, few studies have investigated functional antibody assays in relation to clinical outcomes. In this study we applied a recently developed functional assay of antibody-mediated opsonisation of merozoites, to plasma samples from a longitudinal cohort study conducted in a malaria endemic region of Papua New Guinea (PNG). Phagocytic activity was quantified by flow cytometry using a standardized and high-throughput protocol, and was subsequently evaluated for association with protection from clinical malaria and high-density parasitemia. Opsonising antibody responses were found to: i) increase with age, ii) be enhanced by concurrent infection, and iii) correlate with protection from clinical episodes and high-density parasitemia. Stronger protective associations were observed in individuals with no detectable parasitemia at baseline. This study presents the first evidence for merozoite phagocytosis as a correlate of acquired immunity and clinical protection against *P. falciparum* malaria.

## Introduction

Malaria is caused by the protozoan parasites *Plasmodium spp*., of which *Plasmodium falciparum* is responsible for most of the mortality and morbidity associated with the disease worldwide. There have been major gains in malaria control in endemic countries since 2000, due to measures such as long-lasting insecticidal nets, indoor residual spraying, rapid diagnostic testing, and access to artemisinin combination therapy. While incidence and mortality have declined, malaria still causes 216 million cases and 655,000 deaths per year, predominantly in young children and pregnant women (WHO 2011 report). In addition there are increasing reports of emerging artemisinin drug and insecticide resistance in some regions [1], [2]. Therefore, there remains an urgent need for vaccines to enhance control and contribute to the elimination of malaria [3].

The rationale for vaccine development arose from observations of immunity acquired through natural exposure to malaria. Individuals living in malaria-endemic areas can develop both humoral and cell mediated immunity over time and with exposure (reviewed in [4]). Although this immunity is non-sterilising, it results in reduced parasite densities and protection from life-threatening clinical disease. In particular, transfer of γ-globulin from immune African adults to non-immune children alleviated severe disease [5], [6], which demonstrates the importance of antibodies for clinical protection against *P. falciparum* malaria.

Humoral responses are mounted against pre-erythrocytic, sexual and asexual blood stages of the malaria lifecycle [4], [7], [8]. In particular, merozoite surface antigens are strongly targeted by naturally acquired humoral immunity and hence could serve as targets for vaccine development. At present, the leading blood-stage vaccine candidates with some reported protective efficacy in clinical trials target merozoite proteins [9], [10]. Additional merozoite antigens are currently being investigated as vaccine candidates, and methods for the pre-clinical prioritization of targets are needed. A number of studies have investigated associations between clinical immunity and ELISA-based measures of antibodies to merozoite surface antigens. While antibodies to some antigens such as the MSP3 C-terminus have consistently been associated with protection [11], studies of other vaccine candidates such as AMA-1 and MSP-2 have produced conflicting results [12]–[15]. Serology does not measure affinity, avidity, glycosylation or Fc region status of antibodies, or potentially differing functions of anti-merozoite antibodies such as invasion inhibition and opsonisation. Thus, there is a need to utilise assays that measure functionally protective properties of antibodies.

Opsonising antibodies against merozoites require interactions with neutrophils or monocytes to trigger an anti-parasitic response. Functional *in vitro* assays used to study opsonisation of merozoites include antibody dependent cellular inhibition (ADCI), respiratory burst and phagocytosis assays [16]–[18] ADCI and respiratory burst require the release of soluble mediators which kill parasites or inhibit their growth, while merozoite phagocytosis involves the active removal of merozoites by phagocytic cells following schizont rupture. While the anti-parasitic effector mechanism measured differs between ADCI, respiratory burst and phagocytosis assays, cytophilic IgG and Fcγ receptor-interactions (FcγR) on phagocytes are conserved. This suggests cytophilic antibodies may be able to interact with both monocytes and neutrophils and result in the destruction of opsonised merozoites via multiple effector mechanisms.

To date, merozoite opsonisation assays have been inadequately applied to the study of naturally acquired immunity and disease risk. The ADCI assay has not been rigorously validated for associations with clinical protection in longitudinal cohorts or otherwise, nor has the soluble factor responsible for parasite killing been identified. Antibody-dependent respiratory burst against merozoites has recently been shown to correlate with protection from clinical episodes [19], however the role of high reactive oxygen species (ROS) in malaria is unclear, as ROS production has also been linked to malarial anaemia in children [20]. Antibodies promoting merozoite phagocytosis increase gradually with age, and are higher in individuals resistant to high-density parasitemia [21]. Merozoite surface protein 3 (MSP3) Long Synthetic Peptide, and the MSP3/GLURP fusion protein GMZ2 are candidate vaccines under development. Antibodies to these merozoite vaccine antigens have no direct growth inhibitory function, but produce an ADCI response *in vitro* in the presence of monocytes [22], [23]. Although MSP3 Long Synthetic peptide and GMZ2 vaccines will require phagocyte effector functions to be effective, and have already progressed to clinical trials [10], [24], the potential protective effect of anti-merozoite functional antibodies in general remains to be characterised.

To further investigate the contribution of merozoite opsonising antibodies to naturally acquired immunity to malaria, we applied a recently developed merozoite phagocytosis assay [25] to a longitudinal treatment-reinfection study of semi-immune Papua New Guinean (PNG) children [26]. The results presented here constitute the first demonstration of phagocytosis-inducing opsonising antibodies as a correlate of clinical immunity against *P. falciparum* malaria.

## Materials and Methods

### Study Population and Ethics Statement

Details of the cohort were described elsewhere [26]. In brief, plasma samples were obtained from a prospective treatment-reinfection study of children aged 5–14 years from Madang Province on the north coast of PNG. The presence of *P. falciparum* infection was determined by post-polymerase chain reaction (PCR) ligase detection reaction-florescent microsphere assay (LDR-FMA) and light microscopy (LM), with detection thresholds of approximately >0.3 parasite/µL and >40parasite/µL, respectively [27]. Baseline clinical observations and peripheral blood samples were collected at enrolment. Following enrolment, all participants received 7 days of oral artesunate treatment to clear any current malarial infections. The cohort was followed for 6 months, with symptomatic illness and parasitemia identified with fortnightly follow-ups (active surveillance) and when a child presented with symptoms at the local Mugil Health Centre (passive surveillance). The study was approved by the Medical Research Advisory Committee (MRAC), Papua New Guinea Ministry of Health, The Walter and Eliza Hall Institute Human Research Ethics Committee and the institutional review board of the Veteran’s Affairs Medical Center (Cleveland, Ohio). Written consent was obtained from parents/guardians of all participants.

### Plasma Samples

A venous blood sample was collected at enrolment into heparinised collection tubes, and plasma collected and stored at −80°C. Positive control sera was collected from adults within the Madang province, and negative control sera from malaria naïve anonymous Australian blood donors. For use in phagocytosis assays, plasma samples were serially diluted to a 1/2000 dilution in THP-1 medium.

### THP-1 Monocyte Cell Line

The human monocytic cell line THP-1 (TIB-202, American Type Culture Collection, ATCC) was maintained in RPMI-1640 supplemented with 10% foetal bovine serum (FBS) and 55 µM 2-mercapthoethanol (THP-1 medium), and maintained below a density of 5×10^5^ cells/mL.

### *Plasmodium falciparum* 3D7 Culture and Merozoite Isolation

*P. falciparum* 3D7 parasites were cultured at 4% hematocrit in RPMI-1640 supplemented with 25 mg/mL HEPES, 2 mg/mL sodium bicarbonate, and 10% pooled human serum (parasite medium). Cultures were maintained at 37°C in an atmosphere of 5% CO_2_, 1% O_2_ and 94% N_2_, and synchronized using 5% sorbitol. Late-stage parasites (36–40 hr) were isolated (>95% purity) from uninfected RBCs with a MAC magnet separation column (Miltenyi Biotech). Schizonts were treated with 10 µM E64 (Sigma-Aldrich) for up to 10 hours, and merozoites isolated as previously described [25].

### Phagocytosis Assay

Phagocytosis assays were performed as previously described [25], with minor adjustments. In brief, 1.2×10^6^ EtBr-stained merozoites in 150 µL were mixed with 10 µL of diluted plasma samples, and aliquots of 50 µL were transferred to FBS-coated 96-well U-bottom plates containing 1×10^5^ THP-1 cells to make a final volume of 200 µL per well. Each sample was tested in triplicate. Plates were incubated for 40 minutes at 37°C in 5% CO_2_ humidified incubator, followed by two washes in ice-cold PBS to arrest phagocytosis. Samples were fixed in 2% paraformaldehyde (PFA), and 20,000 events were acquired using a FACSCalibur flow cytometer (BD Bioscience). Data analysis was performed using FlowJo software. Viable cells were gated by forward and side scatter, and phagocytosis positive gates were determined using THP-1 cells alone. Percentage phagocytosis refers to the percentage of EtBr positive cells with immune plasma minus the percentage of positive cells with non-immune plasma.

### Statistical Analysis

Statistical analysis was performed using STATA 9.2 (STATA-Corp, College Station, Texas, USA). Differences in %phagocytosis between categorical variables were assessed using two-sample Wilcoxon rank sum test or Kruskal-Wallis tests. For analysis of associations with clinical outcomes, children with no detectable *P. falciparum* parasitemia by PCR or LM during follow up were regarded as non-exposed, and as such 5% of children were excluded from analysis. In addition, children that failed to clear infections following treatment at baseline(n = 12) as determined by *msp2* genotyping, were also excluded from analysis. A range of demographic and clinical variables were assessed as potential confounders. The median age of individuals was 9.3 years (range, 5–14; interquartile range, 8.1–10.3 years). A range of age groups was investigated, and two groups (<9 years and ≥9 years) were most informative in concordance with previous investigations with this cohort [14], [26], [28]–[31]. Location of residence (attendance at Mugil school and living 1 km from the seaboard) were also included as known confounding variables [26].

To describe optimally associations between opsonising antibody responses and subsequent episodes of *P. falciparum* infection and clinical episodes, phagocytosis responses were rescaled (%phagocytosis_20_) to generate 4 groups reflecting 20% increases (range, original 5–79%; re-scaled: 0.25–3.95). Poisson regression modeling produced incidence rate ratios (IRR) for the incidence of clinical malaria episodes or high-density parasitemia (>5000parasites/µL). Clinical malaria was defined as a measured fever (axillary temperature ≤37.5°C) or history of febrile illness during the 48hours preceding examination in conjunction with *P. falciparum* infection of any density as measured by light microscopy. Eighty percent of the 100 individuals that experienced at least 1 clinical episode had an infection density of greater than 5000 parasites/µL. Seventeen percent of the 92 individuals with an infection of >5000 parasites/µL did not present with clinical symptoms. Cox proportional hazards modeling was used to calculate hazard ratios (HR) for time to first *P. falciparum* infection by PCR, light microscopy, and time to first infection of >500 and >5000 parasites/µL. Although some children had multiple episodes of parasitemia or malaria, HR analysis included time to first re-infection or first clinical episode only.

## Results

### Opsonising Antibody Responses are Robust and Reproducible

**Figure 1:**
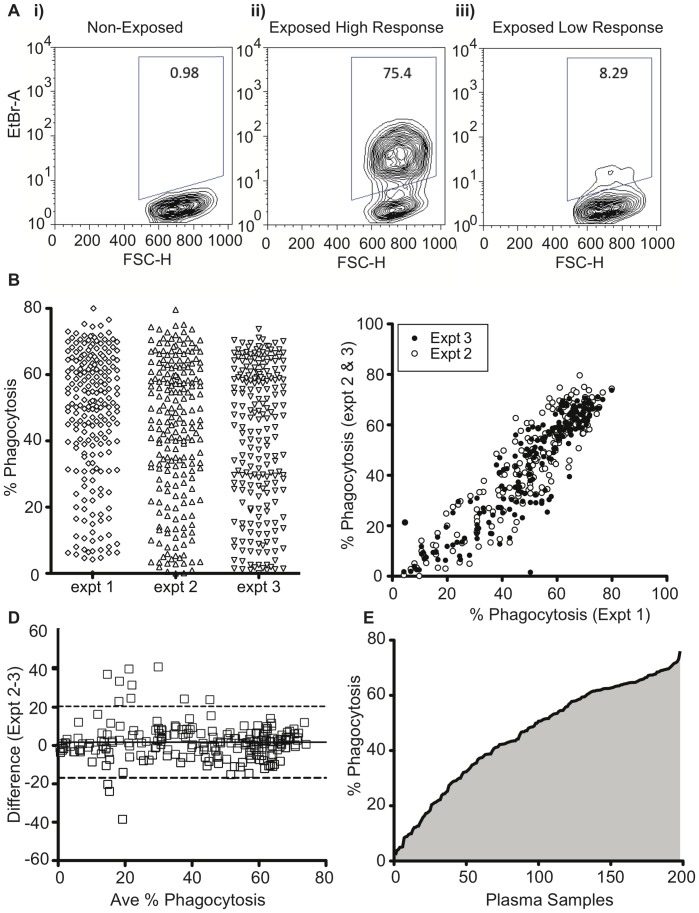
Phagocytosis responses are robust and reproducible. A) EtBr stained merozoites were incubated with plasma, and were added to THP-1 cells. EtBr fluorescence was determined by flow cytometry, and THP-1 cells gated by forward and side scatter. Representative flow cytometry plots show EtBr positive events with i) a pool of non-exposed Australian plasma ii) plasma from an exposed PNG donor that resulted in a high response and iii) PNG donor plasma that resulted in low responses. B) Percentage phagocytosis was calculated for 198 plasma samples from PNG children in three independent experiments. C) Interassay comparisons as determined by Spearman correlation (expt1 vs. expt2 r_s_  = 0.91, p<0.0001; expt1 vs. expt3 r_s_  = 0.93 p<0.0001) and by D) Bland-Altman test (expt1 vs. expt2 bias  = 5.39, sd  = 8.73; expt1 vs. expt3 bias  = 1.79, sd  = 9.3). E) The average % phagocytosis responses from three experiments were ranked by magnitude.

We recently developed a merozoite phagocytosis assay which measures FcγR-dependent phagocytosis responses [25]. When this assay was applied to plasma samples from 198 PNG children, the percentage of THP-1 cells that had phagocytosed merozoites ranged from 5–79% (representative responses in figure 1A). Low background responses below five percent EtBr positive THP-1 cells were observed for non-exposed Australian control plasma, indicating that the assay requires antigen-specific opsonising antibodies. Plasma samples were tested in triplicate, and opsonising responses were measured in three separate experiments (figure 1B). All experiments showed highly correlated measurements (figure 1C, Spearman correlation coefficients; expt1 vs. expt2 r_s_  = 0.91, p<0.0001; expt1 vs. expt3 r_s_  = 0.93 p<0.0001) and highly concordant data (figure 1D, Bland-Altman test; expt1 vs. expt2 bias  = 5.39, sd  = 8.73; expt1 vs. expt3 bias  = 1.79, sd  = 9.3). Therefore, the mean of the three experiments was used for subsequent analyses. Across the 198 plasma samples tested, a spectrum of responses was measured (figure 1e), indicating the assay was of sufficient sensitivity to resolve small differences in opsonising antibody levels between individuals in the cohort.

### Age and Parasitemia at Baseline Strongly Influence Opsonising Antibody Responses

**Figure 2:**
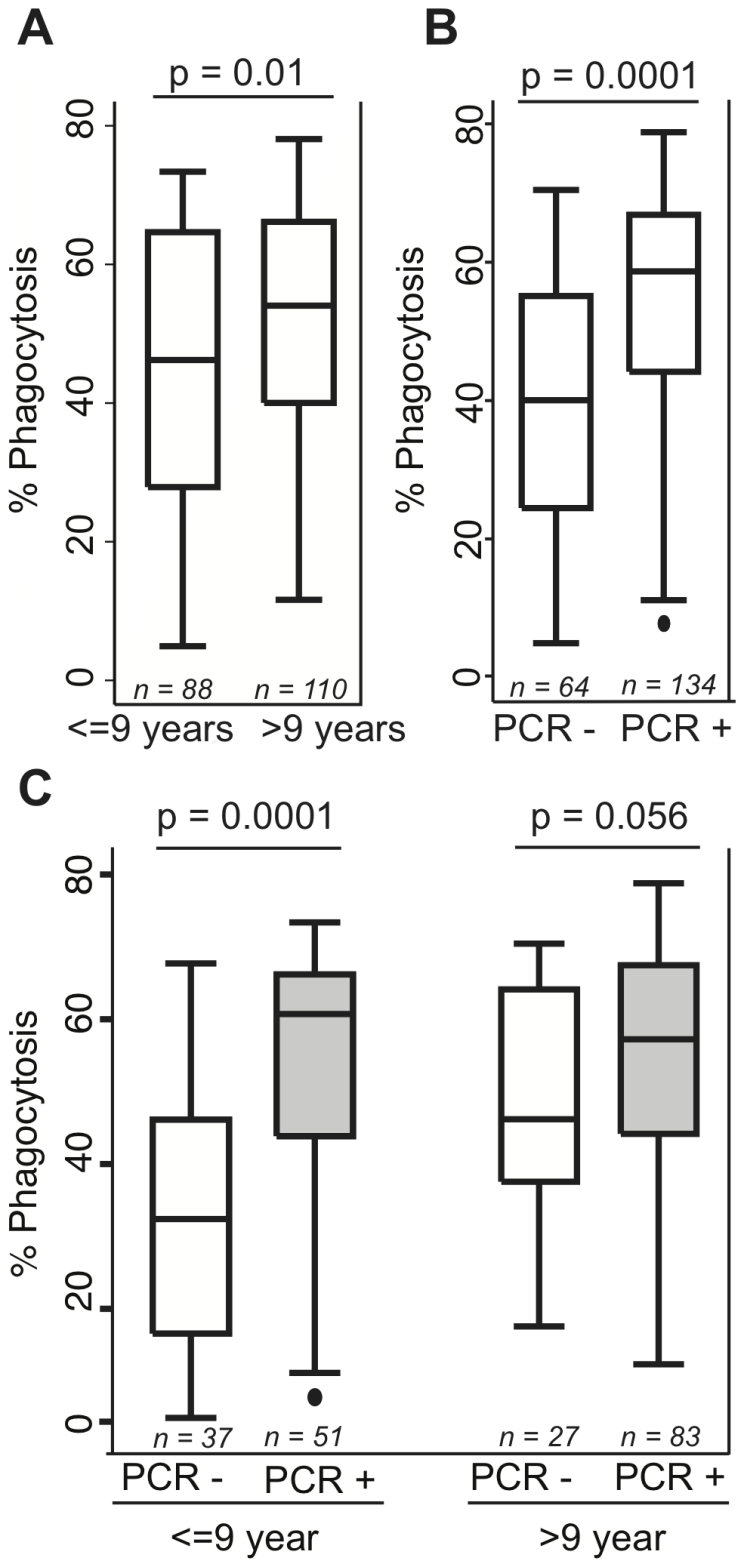
Age and concurrent parasitemia influence opsonising antibody responses. Comparison of opsonising antibody responses in children that were stratified; A) into two age groups of ≤9 years and >9 years; B) on the basis of PCR detectable *P. falciparum* infection at baseline; and C) by both age and infection at baseline. Data are plotted as box-and-whisker plots showing medians and interquartile ranges. Error bars show 95% confidence intervals. Statistical significance was determined by Kruskal-Wallis test (1 d.f.).

Opsonising antibody responses were analysed in relation to a range of clinical and demographic variables. Consistent with previous studies of this cohort, responses were influenced by the age of individuals and the presence of *P. falciparum* parasites at the time of blood sampling [14], [26]. Consistent with previous studies [14], [26], [29], [30], age was most informative when assessed as a binary variable of <9 and ≥9 years of age. The older age group showed higher opsonising antibody responses (median, 54.8; IQR, 40.7–66.9) than younger children (median, 46.2; IQR, 27.9–64.7; p  = 0.01)(figure 2A). As the plasma samples were collected prior to anti-malarial treatment, 68% of individuals had *P.falciparum* infection at baseline as measured by post-PCR LDR-FMA (termed PCR). Parasite positive individuals at baseline displayed increased opsonising responses (median, 58.7; IQR, 44.2–66.9) than those without concurrent infection (median, 40.1; IQR, 24.5–55.1; p  = 0.0001) (figure 2B). When individuals were stratified by age and by parasitemia at baseline, higher opsonising antibody responses in the presence of parasites were only significant in children <9 years (figure 2C). Similar patterns were observed when responses were stratified by the presence or absence of light microscopy positive infections (data not shown). In addition, no differences in opsonising antibodies were observed in PCR positive individuals at baseline for infections above or below the detection limit of light-microscopy (data not shown). Therefore, PCR+ infections showed significant boosting of opsonising antibody responses, particularly in young children.

### Opsonising Antibodies are Associated with Protection from Clinical Malaria and High-density Parasitemia

**Figure 3:**
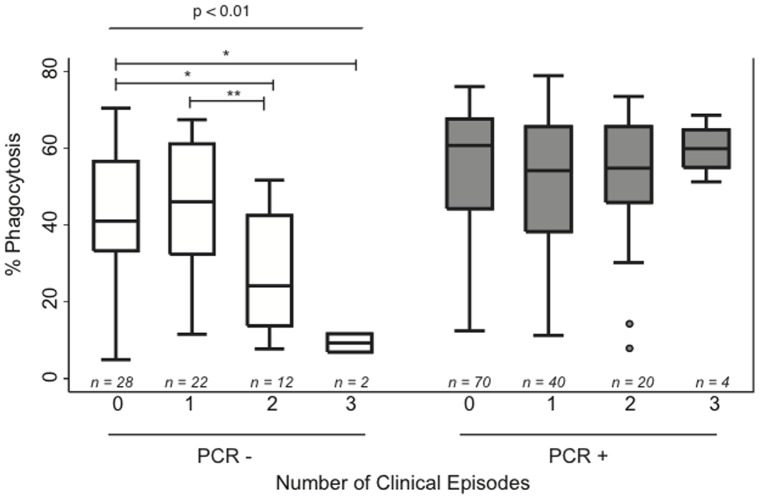
High opsonising responses in individuals protected from clinical malaria, only in the absence of baseline infection. Phagocytic responses were stratified on the basis of PCR detectable *P. falciparum* infection at baseline (white bars represent PCR- individuals and grey bars PCR+ individuals) and the number of clinical episodes (fever ≥37.5°C with parasitemia) during follow-up. Statistical significance was determined by Kruskal-Wallis test (3 d.f., indicated by text) and by Wilcoxon rank sum test (indicated by asterisks, *p<0.05, ** p<0.01).

As highly heterogeneous opsonising antibody responses were observed we investigated whether responses were associated with clinical outcomes and risk of clinical malaria. Higher opsonising antibody responses were observed in individuals who did not experience a clinical episode (median, 56.1; IQR, 39.9–67.0) than in individuals that experienced any number of clinical episodes (median, 49.8; IQR, 31.7–64.0; p  = 0.04). As up to 3 clinical episodes were observed for some individuals, we investigated whether the level of opsonising antibodies differed between those that experienced none, 1, 2 or 3 clinical episodes during follow-up. In addition, we assessed whether baseline parasitaemia influenced the level of opsonising antibody within these clinical groups. When individuals with no detectable parasitemia at baseline were investigated, lower phagocytic responses were observed in individuals who experienced multiple clinical episodes, than in individuals who experienced none or one clinical episode during follow-up (figure 3). Individuals with concurrent infection at baseline showed no differences in phagocytosis responses across the range of clinical episodes.

**Figure 4:**
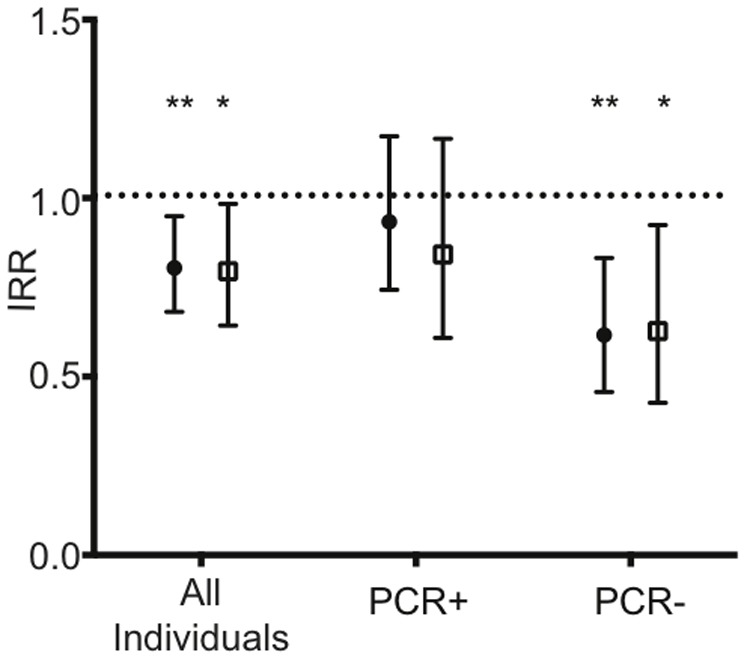
Incidence of clinical malaria episodes during follow-up relative to opsonising antibody responses. Incidence rate ratios (IRR) for %phagocytosis_20_ and the incidence of clinical malaria episodes over the 6 months of follow up were calculated for all individuals and after stratification based on PCR+ *P. falciparum* infection at baseline. Values represent un-adjusted IRR (filled circle) and adjusted IRR (age and location adjusted, open squares) ±95% confidence intervals, and asterisks mark significant associations (* p<0.05,** p<0.01).

To investigate the association between opsonising antibodies and risk of clinical disease, % phagocytosis data was rescaled to represent 4 groups, each a 20% increment in phagocytosis responses. By Poisson regression analysis, %phagocytosis_20_ was associated with a reduced risk of clinical malaria (IRR 0.81; 95%CI, 0.68–0.95; p = 0.01). Interestingly, a significant and stronger association with reduced risk of clinical disease was observed for individuals with no detectable parasitemia at baseline (IRR 0.62; 95%CI, 0.46–0.83; p = 0.002), while parasite PCR+ individuals showed no significant association (IRR 0.94; 95%CI, 0.74–1.173; p = 0.56). The lack of protective association for infection at baseline was maintained when individuals were stratified into PCR+LM− or PCR+LM+ infections at baseline (data not shown). Incidence rate ratios in PCR- individuals remained significant after adjustment for age and location of residence (figure 4). This is equivalent to an age and location-adjusted 38% reduction in risk of clinical disease with each 20% increase in phagocytosis responses.

**Figure 5:**
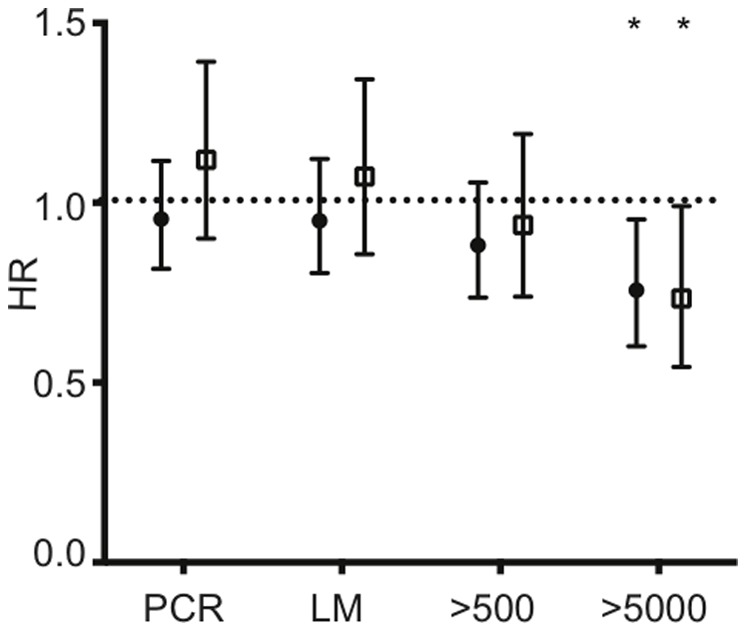
Associations between opsonising antibodies and risk of acquiring *P. falciparum* infections of varying density. Hazard ratios (HR) were generated to assess associations with %phagocytosis_20_ and time to first PCR detectable, light microscopy (LM) detectable, ≥500 parasites/µL, and ≥5000 parasites/µL infections. Observation time was 6 months, and analysis based on first infection only. Values represent un-adjusted HR (filled circle) and adjusted HR (age and location adjusted, open squares) ±95% confidence intervals, and asterisks mark significant associations (* p<0.05).

We then investigated whether opsonising responses were associated with reduced risk of acquiring new *P. falciparum* infections of varying parasite densities. Opsonising antibody responses were not associated with a reduced time to re-infection by PCR, LM, or moderate density infection (>500 parasites/µL). However, a reduced risk of acquiring a high-density infection (>5000 parasites/µL) was observed for opsonising responses (HR, 0.75; 95% CI, 0.60–0.95; p = 0.018). This association was observed without stratification by PCR+ infection, and was unaltered by adjustment for age and location (HR, 0.73; 95% CI, 0.54–0.99; p = 0.04) (figure 5). This equates to an age and location-adjusted 27% reduction in risk of high-density parasitemia with each 20% increase in phagocytosis responses. A slightly lower risk of high-density infection in individuals with no detectable parasitemia at baseline than in those with concurrent infection was observed, but was not significant (data not shown).

## Discussion

Merozoite invasion has been proposed to occur within seconds following schizont rupture [32], casting doubt on the ability of merozoite phagocytosis to be an effective anti-parasite mechanism. However, a new technique for purifying viable merozoites has uncovered the ability of merozoites to remain invasive for ten minutes after mechanical rupture of schizonts [33]. This provides sufficient time for antibodies to opsonise merozoites, and for these merozoites to encounter monocytes or neutrophils in circulation, or macrophages as they pass through the spleen. Therefore, merozoite phagocytosis may represent a significant mechanism contributing to anti-parasite immunity. This is further supported by this study where we newly describe an association between opsonising antibodies leading to merozoite phagocytosis and protection from clinical malaria and high-density parasitemia. These associations remained significant after adjustment for known confounders of age and location of residence. Opsonising antibodies increased with age, but were strongly influenced by concurrent infection. Phagocytic responses were reduced in individuals susceptible to multiple clinical episodes, which suggests that steady state levels of opsonising antibody are a suitable predictor of immune status in children.

Limited data exists on the maintenance of *P. falciparum* antibodies in children, or boosting of immune responses in response to infection. We found that individuals infected with *P. falciparum* at baseline showed increased opsonising antibody levels, an effect observed more strongly in younger children. Fifty per cent of the baseline PCR+ individuals were below the detection limits of light microscopy, which indicates low-density infections (<40 parasites/µL) are enough to boost antibody responses. However, antibody responses to *P. falciparum* antigens are believed to have a dynamic and short-lived profile, particularly in young children or non-immune young adults that are unable to mount an efficient anti-malaria B cell memory response [34]–[36]. Longitudinal studies in African children report the half-lives of merozoite specific antibodies range between 6 to 52 days following malaria infection, with younger children displaying the most rapid decay of antibody responses [34], [36]. In this context it is reasonable to propose that the increased opsonising responses detected in individuals with concurrent infections at baseline reflect short-lived (and presumable low affinity) antibodies elicited in response to those infections.

Interestingly, no association with reduced incidence of clinical episodes and opsonising antibody responses was observed in children who were parasitaemic at baseline. Asymptomatic carriage of *P. falciparum,* as detectible on thick smear by light microscopy, has been reported to confer some protection from clinical episodes [37]. In this study we observed no difference in the level of opsonising antibody or risk of clinical disease in those with light-microscopy positive infections compared to infections that were only PCR positive. In contrast, merozoite opsonising antibody responses in children without parasitaemia at baseline were associated with reduced risk of clinical disease, suggesting a longer-lived immunological status that might more reliably reflect naturally acquired immunity. These findings emphasize the importance of adjusting for baseline parasitological status in investigating correlates of acquired humoral immunity to malaria.

Monocytes purified from immune individuals have been shown to phagocytose merozoites more efficiently than monocytes from non-immune individuals; indicating that monocytes may also be primed for merozoite phagocytosis *in vivo* [17]. Although we did not assess the endogenous monocyte phagocytic capacity in individuals of this study, we demonstrated that opsonising antibody responses were significantly associated with reduced incidence of clinical episodes. While these results do not establish a causative link with protection from clinical disease, mouse models of malaria strongly support the role for anti-merozoite opsonising antibodies. Human monoclonal antibodies to *P. falciparum* MSP1_19_ failed to transfer immunity to mice unless recipients were transgenic for the human FcγRI receptor [38], indicating the importance of phagocyte effector functions for blood stage humoral immunity. Initiation of ADCI requires engagement of FcγRII and FcγRIII [39], while cross-linking of FcγRI is required for respiratory burst [19]. The Fc receptors involved in merozoite phagocytosis remain undefined, but likely mirror the need for FcγRI, and to a lesser extent FcγRII during phagocytosis of *P. falciparum* trophozoites [40] and other pathogens [41], [42]. This implicates phagocytosis, and/or respiratory burst, as the likely effector mechanisms following passive transfer of humoral immunity. ROS release has been considered a downstream event following phagocytosis of opsonised merozoites, however links between the two cellular responses are unclear.

In this same cohort, we have previously shown that *in vitro* IFNγ output from peripheral blood mononuclear cells in response to malaria parasites provides a correlate of cellular immunity to *P. falciparum* [43], [44]. IFNγ potently enhances FcR-mediated monocyte/macrophage phagocytosis and ROS, and may function to promote opsonising immunity to malaria. Cell populations capable of producing IFNγ in response to malaria include γδ Tcells, NK cells, CD1-restricted NKT cells [45], [46] and conventional CD4 and CD8 T cells [47], indicating a wide range of potential cellular interactions across the innate and adaptive immune systems capable of promoting phagocytosis and parasite killing in concert with opsonising antibodies.

Currently, there are vaccines under development that are designed to induce a cytophilic antibody profile and promote FcγR-mediated phagocyte responses [10], [24]. This study describes an association between opsonising antibody responses to *P. falciparum* merozoites with protection from clinical disease and high-density parasitemia, measured in an FcγR-dependent *in vitro* assay. ADCI and respiratory burst assays require the use of primary cells, which may be a factor responsible for the inter-experimental variability and reproducibility issues associated with such assays. The use of a monocytic cell line in this study enabled a highly reproducible readout, and the associations with clinical outcomes supports the application of this functional assay of anti-merozoite immunity to larger cohorts and vaccine trials. The clinical protection observed in this study suggests merozoite phagocytosis may be an important anti-parasitic effector mechanism of natural acquired immunity to *P. falciparum* malaria, thus further investigation into the protective antigenic targets of this response is warranted.
